# Effect of dietary phenolic compounds intake on mortality in the “Seguimiento Universidad De Navarra” (SUN) Mediterranean cohort

**DOI:** 10.1007/s00394-025-03581-5

**Published:** 2025-01-24

**Authors:** Zenaida Vázquez-Ruiz, Estefanía Toledo, Facundo Vitelli-Storelli, Maira Bes-Rastrollo, Miguel Ángel Martínez-González

**Affiliations:** 1 Department of Preventive Medicine and Public Health, University of Navarra, Instituto de Investigación Sanitaria de Navarra (IdiSNA), Pamplona, Spain; 2 Biomedical Research Network Centre for Pathophysiology of Obesity and Nutrition (CIBEROBN), Carlos III Health Institute, Madrid, Spain; 3 Grupo de investigación en Interacciones Gen-Ambiente y Salud (GIIGAS), Instituto de Biomedicina (IBIOMED), University of León, León, Spain; 4 Department of Nutrition, Harvard T. H. Chan School of Public Health, Harvard University, Boston, MA 02115 USA

**Keywords:** Dietary phenolic compounds, Flavonoids, Mortality, SUN cohort

## Abstract

Plant-based dietary patterns have been demonstrated to reduce the risk of non-communicable disease (NCD), including cardiovascular disease (CVD), type 2 diabetes, cancer, and all-cause mortality. Phenolic compounds (PC), abundant in plant-based foods, have been considered as instrumental in this attenuation of NCD risk. We evaluated the association between dietary intake of PC and the risk of all-cause mortality in a relatively young Mediterranean cohort of 18,173 Spanish participants in the “Seguimiento Universidad de Navarra” (SUN) project, after a median follow-up of 12.7 years. Intake of PC was estimated at baseline and repeatedly after 10-year follow-up using a 136-item validated food frequency questionnaire and the Phenol-Explorer database. During 236,329 person-years, 544 deaths were confirmed. Cox regression models compared low intake (lowest quintile) vs. high intake (the four upper quintiles merged, as a reference category) of total energy-adjusted PC intake. Adjusted hazard ratios (HR) for low PC intake among those participants aged over 45 years during follow-up were 1.32; 95% CI 1.02–1.71 for all-cause mortality, HR:1.44; 95% CI 1.02–2.02 for cancer mortality; HR: 0.88; 95% CI 0.47–1.66 for CVD mortality and HR: 1.69 95% CI 1.04–2.74 for causes of death other than cancer or CVD. In conclusion, a low intake of total PC was associated with a significantly higher risk of all-cause mortality, and, specifically, a higher risk for cancer in a relatively young cohort. Among PC classes a low intake of flavonoids and phenolic acids showed significant effects for non-cancer/non-CVD mortality. Cherries, chocolate, apples and pears, olives, and coffee, were the major sources of between-person variability for total PC intake in our Mediterranean cohort.

**Supplementary Information:**

The online version contains supplementary material available at 10.1007/s00394-025-03581-5.

## Introduction

According to World Health Organization (WHO) non-communicable diseases (NCDs) collectively accounted for 74% of deaths globally in 2019 and are on the rise [1]. Cardiovascular diseases (CVD), cancer, chronic respiratory disease, and diabetes account for more than 80% of all premature deaths from NCDs. In Spain, according to the National Statistics Institute, the leading causes of death in 2022 were circulatory system diseases (26.0%), cancer (24.8%) and respiratory diseases (9,3%) [2]. Particularly, among adults aged between 40–79-year-old, cancer (40.2%) and circulatory system diseases (20.4%) were the main causes of death. High blood pressure, increased blood glucose, overweight or obesity, and hyperlipemia are the main metabolic risk factors that contribute to the increase of NCDs worldwide [3]. These factors are closely related to unhealthy diets which along with other lifestyle behaviors such as smoking or sedentary lifestyles lead to an increased risk of death from NCDs [4].

Among eight dietary patterns examined by Wang et al. in 205,852 U.S. health professionals followed for 32 years, those which reflect low insulinemic, low inflammatory and diabetes risk-reducing diets may confer the largest risk reduction for various NCDs. Each of the analyzed dietary patterns included the consumption of fruits and vegetables as a common component in their scores [5]. Several studies have shown that a plant-based dietary pattern (also named as a “provegetarian” dietary pattern) that emphasized high-quality foods reduced the risk of CVD, type 2 diabetes, cancer, and all-cause mortality [6]. Due to the abundance of phenolic compounds (PC) in plant-based foods and beverages, significant research has been conducted on the antioxidant and anti-inflammatory properties of PC as adjuvants in the attenuation of NCD risk factors [7]. These molecules are synthetized by plants as secondary metabolites, under unfavorable conditions such as the presence of pathogens or adverse climatic conditions [8]. PC are found in widely consumed foods such as extra-virgin olive oil, grapes, apples, berries, citrus fruits, nuts and legumes and in popular drinks such as coffee, tea or wine [9].

Flavonoids and flavonoids subclasses are the most extensively studied subclasses of PC and a high consumption of these compounds seems to lower mortality [10, 11]; however, other studies have found a less clear relationship between some classes of PC consumption and mortality [12, 13]. On the other hand, some potential adverse effects have been reported on the consumption of high doses of certain PC, especially when they are consumed in pharmacological doses as a dietary supplement [14]. The state of the intestinal microbiota, health status, bioavailability and pharmacokinetics of different PC may affect the observed results [15].Therefore, it seems crucial to keep researching how PC intake may affect various health outcomes.

In this context, Mediterranean populations, who have traditionally followed a preferentially plant-based diet or a provegetarian diet with a high intake of PC-rich foods, may provide a solid foundation for determining whether low PC intake can play a detrimental role in mortality risk, and in the risk of dying due to different causes. Most of the evidence has been focused on flavonoid intake, but we consider that understanding the effect of total PC and of each class of PC intake on overall and cause-specific mortality can help to better elucidate the role of each of these groups. Our findings might help develop evidence for setting intake targets and optimizing diets through plant-based foods.

Recently, a study in a Spanish population showed protective effects against mortality of some PC subclasses intake considering information on diet at baseline [16]. The present study aims to analyze diet using repeated dietary measurements both at baseline and after 10 years follow-up in a well-known Mediterranean cohort and analyze the effect of low intake of total PC and specific PC classes on overall and cause -specific mortality. We consider that studying the roles not only of total PC intake but also by specific subclasses may represent a more solid approach for capturing the long-term effects of PC usual consumption. The SUN cohort has been specifically designed to assess diet-disease relationships in a Mediterranean context and diet was evaluated using a validated food frequency questionnaire (FFQ), which may improve comparability with other large cohort studies.

## Materials and methods

### Study population

**Fig. 1:**
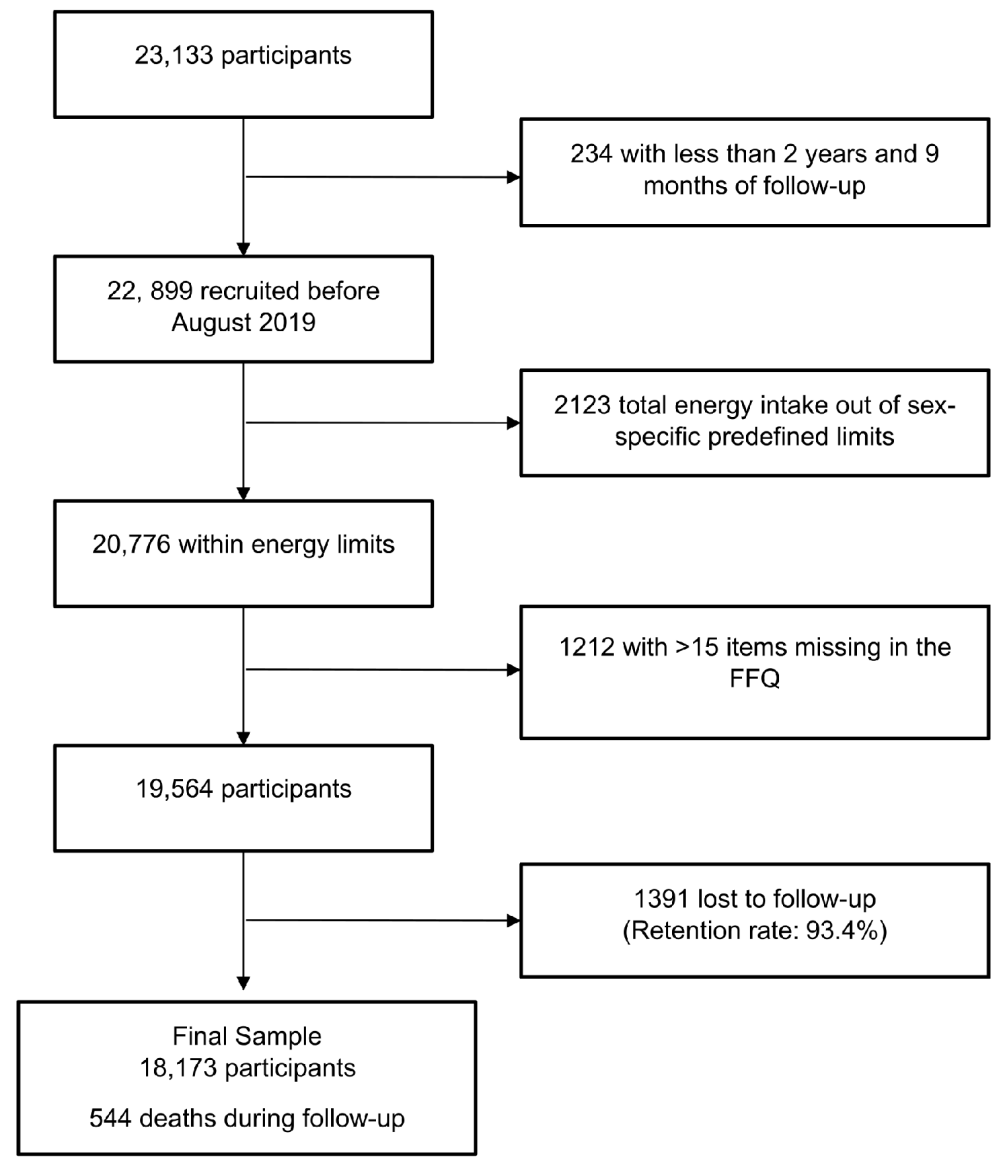
Flow-chart of study participants

The SUN Project is an ongoing, multi-purpose cohort of Spanish university graduates that started recruiting in December 1999 and is permanently open. As by May 2022, 23,133 Spanish university graduates had been recruited. A more detailed description of the design and methodology of this cohort is available elsewhere [17]. In summary, self-administered questionnaires collecting data on sociodemographic characteristics, health status, family history of disease, physical activity, and other lifestyle information were sent by mail or electronically at the start of the study. Follow-up questionnaires are sent every two years and gather updated data on lifestyle, dietary behaviors, medication use and health outcomes. For the present analyses participants with insufficient follow-up (less than 2 years and 9 months) and lost to follow-up were excluded, as well as those with total energy intake outside the predefined limits (< 500 or > 3,500 kcal/d in women, or < 800 or > 4,000 kcal/d in men) [18]. We also excluded participants with > 15 missing items in the FFQ. Finally, a total of 18,173 participants were included in this analysis, with a mean follow-up time of 12.7 years (SD = 5.6 years) and a retention rate of 93.4% (Fig. 1). This study was carried out in conformity with the Declaration of Helsinki and the protocol (including the informed consent process) and was approved by the Institutional Review Board of the University of Navarra. Completion of the self-administered questionnaire was considered as constituted informed consent.

### Assessment of dietary phenolic compounds intake

At the time of study enrollment and at a 10-year follow-up, dietary intake was evaluated using a previously validated semi-quantitative FFQ [19, 20] with 136 food items. The FFQ comprised nine intake consumption options for each food item (never or seldom; 1–3 times/month; once/week; 2–4 times/week; 5–6 times/week; once/day; 2–3 times/day; 4–6 times/day; and > 6 times/day). To determine the daily intake of nutrients and energy, the standard portions were multiplied by the consumption frequency of each food and by the nutritional content or kilocalories of energy according to the Spanish food composition Table [21]. Adherence to the Mediterranean dietary pattern was assessed with the score (0–9 points) described by Trichopoulou et al. [22].

The PC content of each food was determined using the Phenol-Explorer database version 3.6 (www.phenol-explorer.eu) [23]. When more than one food was included in an FFQ food record, a weighted average was calculated using the average dietary consumption of the adult Spanish population [24]. The most common extraction method used for estimating PC intake was high-performance liquid chromatography (HPLC). Normal-phase HPLC was used to estimate proanthocyanidins content. To calculate the aglycone content for lignans and phenolic acids in specific foods such as cereals, olives, and beans, data from HPLC after the hydrolysis were used if available. Total and by class PC intake (milligrams per day) was calculated using the sum of all individual PC for each food record in the FFQ. In addition, the proportion of each meal or type of food that contributed to total PC intake was calculated. Total PC intake showed good correlations with estimated intakes using our FFQ [25] and some validation studies also have demonstrated directly or indirectly the validity of a FFQ in estimating PC intake [26].

### Ascertainment of deaths

The primary outcome was all-cause death. Through active and ongoing contact with participants, information on death and its causes was gathered. More than 85% of the deaths were identified based on reports from family, coworkers, and the postal service. We investigated medical data with the approval of relatives to confirm the fatalities. For the rest of the deaths, we checked the Spanish National Death Index—a central index that collects information on any death registered in Spain—at least once a year to confirm the vital status of all participants and to identify their cause of death if it was unknown. The most recent check on mortality and its causes was done in January 2022. Death certificates and medical records detailing the reasons of death were thoroughly collected.

### Assessment of covariates

At baseline, participants reported information on potentially confounding factors, such as socio-demographic characteristics (sex, age, level of education and marital status), validated anthropometric measures (weight and height) [27], smoking status, sedentarism, leisure time physical activity (METs-h/week) [28], use of medication and other health related behaviors. At the beginning of the study, details regarding their personal and family members’ medical history were also collected. The validity of self-reported medical data was validated in the SUN cohort [29, 30].

### Statistical analysis

Total and by class PC intake was adjusted for total energy intake using Willett’s residuals method separately form men and women [18] and grouped into quintiles. Descriptive statistics were used to analyze participants’ baseline information adjusted for age and sex by the inverse probability weighting method, using proportions or means (and standard deviations, SD) according to quintiles of PC intake. Crude and multivariable time-dependent Cox regression models were used to estimate hazard ratios of all-cause mortality across quintiles of total and by class PC intake considering age as the underlying time variable and assuming the four upper quintiles as the reference category. The rationale for this decision was that, after considering the abundant customary content of PC in the Mediterranean diet, we focused our analyses on assessing the potential detrimental effect of a low intake by using the 4 upper quintiles as the reference category and assessed hazard ratios (HRs) with their 95% confidence interval (CI) for the lowest quintile. Therefore, we assumed an L-shaped association, with an expected plateau for successively increasing categories of high intake. Time at entry was defined as the date of completion of the first questionnaire and time at exit as the date of death or date at which participants completed their last follow-up questionnaire, whichever came first. Multivariable adjusted model 1 was stratified by age (five-year periods), recruitment period, marital status, and years of university education, and adjusted for energy intake (kcal/day), smoking status (never smoker, current smoker or former smoker), lifetime tobacco exposure, passive smoking, body mass index (BMI) (kg/m^2^) (continuous and its quadratic term), height, prevalent diseases (cardiovascular disease, diabetes, cancer, atrial fibrillation, dyslipidemia, hypertension and depression) family history of disease(cancer, and CVD), physical activity (METs-h/week) and TV watching (hours/day).

Repeated measurements analyses were performed on those participants who completed a second FFQ after 10-year follow-up using cumulative averages to reduce the effect of dietary variations between baseline and 10 years of follow-up. We did not update diet if participants reported a diagnosis of cancer, CVD or diabetes during follow-up since participants might have change their usual diet after the diagnosis of a major chronic diseases (*n* = 909 for total sample and *n* = 652 in participants over 45 years). HRs were estimated for subjects over 45 years old during follow-up. This cut-off point was taken according to the mortality data by age provided by the National Institute of Statistics in 2021. The highest increase in cancer mortality occurs from the age of 40 years onwards, however, looking at CVD mortality, the higher risk is observed from the age of 50 years on, so we decided to take the midpoint, 45 years as these were the main causes of mortality in our cohort [31].

Tests of linear trend were applied for the evaluation of dose–response relationships across quintiles, assigning to each category of the total intake its quintile-specific median and using the resulting variable as continuous in the abovementioned models. Furthermore, flexible regression models (cubic splines) were performed to better understand the relationship between PC intake and total mortality. All analyses were repeated for each PC class. Pearson correlation coefficients between total PC and each subgroup at baseline and at 10 years of follow-up were calculated. The contribution of the main food sources to total PC intake and by class was calculated. Between-person variability in total PC intake and by class was calculated by conducting nested least-squares linear regression models after stepwise selection regression.

We also performed sensitivity analyses to explore the robustness of our findings by further adjusting for the intake of PC not belonging to the analyzed class and excluding external causes of mortality. Interactions between total PC intake and BMI or sex were also explored (a priori decisions) using likelihood ratio tests. No other interactions were evaluated.

All analyses were performed with the Stata statistical software package v.17 (College Station, TX, USA; Stata Corp LLC) with the SUN database updated in December 2022. All *p*-values presented are two-tailed and the statistical significance was set at 0.05.

## Results

Participants’ mean baseline age was 37.7 years (SD = 12 years), and the mean BMI was 23.5 kg/m^2^ (SD = 3.5 kg/m^2^). Table 1 shows the main baseline characteristics of the 18,173 of participants by quintiles of baseline energy-adjusted PC intake. Participants in the upper quintile of PC intake showed the highest percentage of women (62.9%), and of married subjects, and presented higher values of leisure time physical activity. This upper quintile also presented higher percentages of energy provided by carbohydrates, the highest fiber and alcohol intakes, and the best adherence to the Mediterranean diet score^32^. On the other hand, participants in the first quintile of PC intake showed the highest percentage of energy provided by fat. Other participants´ conditions such as current smoking, BMI, hypertension, depression, and CVD at baseline showed similar values across quintiles (Table 1).

**Table 1:** Baseline characteristics* of participants across sex-specific energy-adjusted quintiles of phenolic compounds dietary intake

		Energy-adjusted quintiles of total phenolic compounds intake				
		Q1 (*n* = 3565)	Q2 (*n* = 3565)	Q3 (*n* = 3564)	Q4 (*n* = 3565)	Q5 (*n* = 3634)
Age (years)†		39 (13.7)	37.9 (12.5)	37.7 (11.9)	37.8 (11.7)	37.9 (12)
Sex (female))†		55.9%	59.9%	61.8%	61.9%	62.9%
Total polyphenol (mg/day)		416.8 (98.7)	603.4 (39.8)	735.7 (39.1)	893.5 (55.9)	1280.7 (328.9)
Flavonoids (mg/day)		204.4 (86.2)	315.5 (78.7)	397.6 (91.5)	498.3 (117.3)	774.8 (313.2)
Lignans (mg/day)		1.6 (0.6)	1.9 (0.6)	2.2 (0.7)	2.4 (0.8)	2.9 (1.3)
Phenol acids (mg/day)		182.9 (70.3)	250.4 (74.7)	294.8 (86.0)	346.2 (107.6)	445.5 (181.8)
Stilbenes (mg/day)		0.6 (1.2)	1.0 (1.7)	1.2 (2.2)	1.5 (2.7)	1.9 (3.9)
Other polyphenols (mg/day)		27.2 (19.1)	34.6 (19.9)	39.9 (21.9)	45.1 (27.6)	55.5 (51.6)
Body mass Index (kg/m^2^)		23.6 (3.6)	23.6 (3.5)	23.6 (3.5)	23.6 (3.5)	23.3 (3.5)
Physical activity (METS-h/week)		18.7 (21.4)	19.7 (20.6)	22.1 (22.6)	23.4 (23.1)	26.3 (27.4)
University education (years)		5.0 (1.5)	5.1 (1.5)	5.1 (1.6)	5.1 (1.5)	5.0 (1.5)
TV watching time (hours/day)		1.7 (1.3)	1.6 (1.2)	1.6 (1.1)	1.6 (1.1)	1.5 (1.2)
Smoking						
Current smoker		23.6%	22.6%	22.6%	23.6%	22.3%
Former smoker		26.0%	26.7%	29.3%	28.6%	29.8%
Never smoker		49.7%	50.0%	47.6%	47.1%	47.2%
Cumulative pack-years of smoking		6.7 (11.5)	5.8 (9.7)	6.1 (9.9)	6.1 (9.6)	6.4 (10.0)
Marital status (married)		38.2%	46.5%	52.7%	55.1%	58.5%
Dyslipidemia at baseline		17.7%	17.7%	19.5%	20.3%	20.8%
Hypertension at baseline		11.8%	10.9%	11.0%	10.2%	10.7%
CVD at baseline		1.4%	1.5%	1.6%	1.3%	1.6%
Atrial fibrillation at baseline		0.4%	0.6%	0.5%	0.7%	0.6%
Diabetes at baseline		2.3%	1.4%	1.8%	2.3%	1.8%
Depression at baseline		12.2%	10.3%	11.4%	11.2%	12.2%
Cancer at baseline		2.3%	2.7%	2.6%	3.0%	2.4%
Family history of CVD		7.4%	7.9%	7.1%	7.6%	8.6%
Family history of cancer		8.7%	8.8%	8.4%	8.4%	8.6%
Total energy intake (Kcal/day)		2574 (571)	2289 (584)	2233 (594)	2295 (594)	2469 (615)
Carbohydrate intake (% energy)		42.5 (7.5)	42.3 (6.9)	42.9 (6.8)	44.0 (6.8)	46.2 (7.6)
Protein intake (% energy)		18.0 (3.2)	18.6 (3.1)	18.7 (3.1)	18.5 (3.2)	17.7 (3.1)
Fat intake (% energy)		37.9 (6.6)	37.3 (6.0)	36.4 (5.9)	35.3 (6.0)	33.9 (6.6)
Dietary fiber intake (g/day)		19.7 (7.4)	19.8 (7.5)	21.3 (7.8)	23.8 (8.7)	29.6 (12.1)
Alcohol intake (g/day)		5.6 (9.8)	6.0 (8.5)	6.4 (9.0)	7.2 (10.2)	7.8 (11.9)
Vitamin and/or mineral supplements intake (%)		17%	17%	18%	19%	24%
Adherence to MDS (0–9 score)		3.4 (1.6)	3.8 (1.6)	4.2 (1.7)	4.7 (1.7)	5.1 (1.7)

* Adjusted for inverse probability weighting for sex and age. Values are expressed as means and standard deviations or percentage. MET metabolic equivalents; SUN, Seguimiento Universidad de Navarra; CVD Cardiovascular disease; MDS, Mediterranean Diet Score proposed by Trichopoulou et al. †Not adjusted

The energy-adjusted mean ± SD of total PC intake was 784.9 ± 335 mg/day, of which 55.5% were provided by flavonoids (435.8 ± 273 mg/day) mainly by chocolate (23.3%) and apples and pears (13.2%). Phenolic acids were the second main contributor to total PC intake, 38.8% (305 ± 153.4 mg/day) mainly provided by regular coffee (29,2%) and decaffeinated coffee (11,3%). Other phenolic compounds subclasses provided 5% of total PC intake (40.9 ± 33.7 mg/day) mainly through olives (43.9%) and coffee (11.4%). Lignans intake (2.2 ± 1.06 mg/day) was mostly provided by carrots and pumpkins (12.4%) and olive oil (11.4%), and finally the primary source of stilbenes (1.24 ± 2.6 mg/day) was red wine (89.4%), (Table 2). Cherries, chocolate, apples and pears, coffee and olives, were among the major contributors to total PC intake accounting for most of the between-person variability in total PC intake. Chocolate and fruits were the main contributors of variability for flavonoids; vegetables and olive oil for lignans; coffee, carrots, pumpkins, olives and cherries for phenolic acids, and red wine for stilbenes (Table 2).

**Table 2:** Main sources of between-person variability and their contribution to the total intake of phenolic compounds and their classes (cumulative R2 and change in R2)

	Cumulative *R*^2^	Change in *R*^2^	Contribution (%)
Total phenolic compounds			
Cherries	0.244		7.7
Chocolate	0.481	0.237	13.1
Apples and pears	0.614	0.133	8.9
Coffee	0.664	0.052	8.9
Olives	0.735	0.069	11.4
Flavonoids			
Chocolate	0.436		23.3
Cherries	0.666	0.229	5.9
Apples and pears	0.804	0.138	13.2
Oranges	0.858	0.053	7.5
Orange juice	0.888	0.029	6.7
Lignans			
Broccoli and cabbage	0.772		7.7
Carrot and pumpkin	0.835	0.062	12.4
Olive oil	0.895	0.06	11.4
Tomato	0.952	0.57	9.1
Orange, grapefruit, and tangerine	0.98	0.028	7
Phenolic acids			
Coffee	0.279		29.2
Decaffeinated coffee	0.571	0.292	11.3
Carrot and pumpkin	0.685	0.113	8.1
Olives	0.775	0.09	7.5
Cherries	0.855	0.079	5.7
Stilbenes			
Red wine	0.993		89.4
Other wines	0.998	0.005	3.5
Grapes	0.999	0.001	3.5
Other phenolic compounds			
Olives	0.772		43.9
Olive oil	0.835	0.062	20.1
Breakfast cereal	0.895	0.06	8.5
Whole-grain bread	0.952	0.057	8.2
Orange juice	0.98	0.028	7.6

Cumulative R^2^ values were determined from nested regression analyses after stepwise selection

Among 236,329 person-years of follow-up 544 deaths were confirmed during a mean follow-up time of 12.7 years (Fig. 1). Across quintiles of total PC intake by class a significant inverse linear trend across quintiles of energy-adjusted flavonoid intake and all-cause mortality was observed when analyses were restricted to participants older than 45 years in multivariable regression model 1 (p for trend: 0.01) and in time-dependent Cox models using repeated measurements of diet (p for trend: 0.02), (Table 3). When we included participants younger than 45 years, we did not find any significant linear trend in multivariable regression models (Supplementary Table 1).

Table 4 shows risks of all-cause and cause-specific mortality in participants older than 45 years comparing a low intake versus moderate-high intake (Q1 vs. Q2-Q5 which was the reference category) of total PC and by class. In multivariable Cox regression model 1, a low total PC intake was associated with a significantly increased risk of all-cause mortality based only on baseline intake (HR: 1.33; 95% CI 1.02–1.76; *p*-value: 0.03) and a similar reduction was observed when incorporating cumulative averages using repeated measurements after 10 years of follow up (HR: 1.32; 95% CI 1.02–1.71; *p*-value: 0.03). For cancer mortality a low PC intake (lowest quintile) was found significantly associated with higher risk of death when we compare Q1 vs. Q4-Q5 with repeated measurements (HR: 1.44; 95% CI 1.02–2.02; *p*-value: 0.03). For CVD mortality, we observed non-significant association for a low intake of total PC in both model 1 (HR: 0.81; 95% CI 0.41–1.62) and using cumulative averages of PC intake (HR: 0.88; 95% CI 0.47–1.66). Most statistically significant results were found for PC intake on other (*n* = 135) and unknown causes (*n* = 12) of mortality. In this subgroup of non-cancer, non-CVD deaths, the main causes of death, were respiratory (*n* = 49) and neurological disease (*n* = 29), which together accounted for 48.3% of deaths in this category. Unknown mortality refers to deaths whose causes have not yet been identified. A low total PC intake showed a considerably higher risk of non-cancer/non-CVD deaths, both in model 1 (HR: 1.67; 95% CI 1.02–2.74; *p*-value: 0.04) and in repeated measurements analysis using cumulative average intake (HR: 1.69; 95% CI: 1.04–2.74; *p*-value: 0.03). When we included participants under 45 years old in the analysis, low intake of total PC did not show significant results in all-cause nor in cause-specific mortality (Supplementary Table 2).

**Table 3:** Hazard ratio (HR) and 95% confidence intervals (CI) of all-cause mortality cases according to quintiles of phenolic compounds intake and classes restricted to participants aged over 45 years during follow-up, using the lowest quintile as the reference category

		Quintiles of intake among participants aged over 45 years					
		1	2	3	4	5	*p* for trend
**Total phenolic compounds**							
Median intake (mg/d)		434.9	603.8	734.2	888.2	1181.9	
N		3635	3635	3634	3634	3655	
Cases		67	88	90	101	140	
Person-years		14,915	20,099	22,925	25,662	28,748	
Age and sex adjusted HR (95% CI)		1 (ref.)	0.76 (0.56–1.01)	0.76 (0.57–1.02)	0.70 (0.52–0.93)	0.75 (0.57–0.98)	0.14
Multivariable adjusted model 1		1 (ref.)	0.77 (0.55–1.08)	0.79 (0.57–1.10)	0.71 (0.51–0.99)	0.74 (0.53–1.02)	0.12
Repeated measurements model 1		1 (ref.)	0.76 (0.54–1.06)	0.79 (0.56–1.10)	0.70 (0.56–0.98)	0.77 (0.57–1.06)	0.27
**Flavonoids**							
Median intake (mg/d)		191.8	302.4	388.9	499.0	722.5	
N		3635	3635	3634	3634	3655	
Cases		75	85	97	112	117	
Person-years		16,950	20,276	23,733	25,028	26,362	
Age and sex adjusted HR (95% CI)		1 (ref.)	0.81 (0.61–1.08)	0.89 (0.67–1.18)	0.72 (0.54–0.96)	0.67 (0.50–0.89)	0.01
Multivariable adjusted model 1		1 (ref.)	0.72 (0.52–1.02)	1.04 (0.75–1.44)	0.76 (0.54–1.05)	0.65 (0.47–0.90)	0.01
Repeated measurements model 1		1 (ref.)	0.80 (0.58–1.12)	0.97 (0.68–1.35)	0.73 (0.52–1.02)	0.68 (0.49–0.95)	0.02
**Lignans**							
Median intake (mg/d)		1.2	1.7	2.1	2.5	3.3	
N		3635	3635	3634	3634	3655	
Cases		70	83	101	114	118	
Person-years		16,147	21,779	22,952	24,999	26,473	
Age and sex adjusted HR (95% CI)		1 (ref.)	0.93 (0.69–1.27)	0.98 (0.73–1.32)	0.94 (0.70–1.27)	0.73 (0.53–0.99)	0.09
Multivariable adjusted model 1		1 (ref.)	0.94 (0.68–1.32)	1.07 (0.77–1.49)	0.91 (0.65–1.27)	0.83 (0.60–1.17)	0.23
Repeated measurements model 1		1 (ref.)	1.01 (0.71–1.41)	0.97 (0.69–1.35)	0.97 (0.70–1.36)	0.73 (0.51–1.03)	0.10
**Phenolic acids**							
Median intake (mg/d)		147.5	222.4	286.5	353.0	484.6	
N		3635	3635	3634	3634	3655	
Cases		62	80	104	107	133	
Person-years		14,413	21,107	21,990	25,510	29,329	
Age and sex adjusted HR (95% CI)		1 (ref.)	0.79 (0.59–1.06)	1.14 (0.86–1.51)	0.86 (0.64–1.16)	0.93 (0.70–1.22)	0.93
Multivariable adjusted model 1		1 (ref.)	0.84 (0.60–1.19)	1.16 (0.84–1.61)	0.95 (0.68–1.33)	0.91 (0.65–1.28)	0.67
Repeated measurements model 1		1 (ref.)	0.84 (0.60–1.17)	1.18 (0.85–1.63)	0.9 (0.6–1.17)	0.91 (0.65–1.28)	0.85
**Stilbenes**							
Median intake (mg/d)		-0.0	0.1	0.4	0.8	3.6	
N		3635	3635	3634	3634	3655	
Cases		73	60	71	96	186	
Person-years		16,932	17,198	20,679	23,720	33,820	
Age and sex adjusted HR (95% CI)		1 (ref.)	0.84 (0.61–1.14)	0.82 (0.61–1.11)	0.90 (0.67–1.20)	1.00 (0.77–1.3)	0.61
Multivariable adjusted model 1		1 (ref.)	0.91 (0.63–1.31)	0.95 (0.66–1.38)	0.95 (0.67–1.35)	1.01 (0.73–1.38)	0.66
Repeated measurements model 1		1 (ref.)	0.99 (0.68–1.43)	0.96 (0.66–1.41)	1.06 (0.76–1.52)	1.01 (0.72–1.40)	0.62
**Other phenolic compounds**							
Median intake (mg/d)		14.1	25.3	34.1	45.2	72.6	
N		3635	3635	3634	3634	3655	
Cases		97	82	88	96	123	
Person-years		20,471	21,996	22,346	22,301	25,235	
Age and sex adjusted HR (95% CI)		1 (ref.)	0.85 (0.64–1.14)	0.93 (0.70–1.23)	0.97 (0.73–1.29)	0.98 (0.75–1.28)	0.63
Multivariable adjusted model 1		1 (ref.)	0.79 (0.56–1.12)	0.97 (0.70–1.34)	1.04 (0.75–1.44)	1.02 (0.74–1.38)	0.95
Repeated measurements model 1		1 (ref.)	0.88 (0.63–1.25)	0.94 (0.67–1.32)	1.03 (0.74–1.45)	1.04 (0.76–1.42)	0.41

Abbreviations: CI, confidence interval; HR, hazard ratio; Q, quintile. All Cox regression models used age as the underlying time variable and were also stratified by age (five-year periods), recruitment period, marital status, and years of university education. Multivariable adjusted model 1: Additionally adjusted for energy intake (kcal/day), smoking status (never smoker, current smoker or former smoker), lifetime tobacco exposure (packs-years), passive smoking (yes/no), BMI (kg/m^2^) and the quadratic term, height (m), prevalent cardiovascular disease, cancer, diabetes, atrial fibrillation, dyslipidemia, hypertension and depression, (yes/no), family history of CVD and cancer (yes/no), physical activity (metabolic equivalents-h/week) (tertiles), TV watching time (hours/day). Repeated measurements analyses were adjusted for the same variables as model 1 with updated data on dietary variables at 10 years of follow-up (except for participants with a diagnosis of cancer, CVD or diabetes during follow-up, *n* = 652)

**Table 4:** Hazard ratio (HR) and 95% confidence intervals (CI) of all-cause and cause-specific mortality according to a low intake (Q1) vs. a moderate-high intake (Q2-Q4) restricted to participants over 45 years old, using the four upper quintiles as the reference category

	All-cause mortality	Cancer mortality	CVD mortality	Non-cancer, non-CVD mortality
Number of cases/person-years	486/112,349.062	243/110,568.956	96/109,624.63	147/107,770.198
**Total phenolic compounds**				
Age and sex adjusted HR (95% CI)	1.34 (1.07–1.68)	1.44 (1.05–1.97)	0.94 (0.53–1.66)	1.33 (0.88–2.01)
Multivariable adjusted model 1	1.33 (1.02–1.76)	1.39 (0.98–1.98)	0.81 (0.41–1.62)	1.67 (1.02–2.74)
Repeated measurements model 1	1.32 (1.02–1.71)	1.44 (1.02–2.02)	0.88 (0.47–1.66)	1.69 (1.04–2.74)
**Flavonoids**				
Age and sex adjusted HR (95% CI)	1.30 (1.04–1.63)	1.33 (0.96–1.84)	1.01 (0.57–1.77)	1.49 (0.99–2.23)
Multivariable adjusted model 1	1.29 (0.99–1.69)	1.30 (0.90–1.85)	0.84 (0.41–1.64)	1.73 (1.06–2.82)
Repeated measurements 1	1.28 (0.98–1.67)	1.32 (0.93–1.87)	0.67 (0.35–1.38)	1.78 (1.09–2.94)
**Lignans**				
Age and sex adjusted HR (95% CI)	1.11 (0.87–1.42)	1.66 (0.75–1.51)	1.08 (0.61–1.89)	1.11 (0.70–1.75)
Multivariable adjusted model 1	1.07 (0.21–1.40)	1.05 (0.72–1.53)	0.96 (0.50–1.87)	1.18 (0.71–1.97)
Repeated measurements model 1	1.09 (0.83–1.44)	1.29 (0.90–1.84)	1.12 (0.60–2.12)	1.08 (0.64–1.83)
**Phenolic acids**				
Age and sex adjusted HR (95% CI)	1.08 (0.86–1.36)	0.93 (0.66–1.32)	0.84 (0.47–1.49)	1.41 (0.95–2.11)
Multivariable adjusted model 1	1.03 (0.80–1.36)	0.89 (0.61–1.31)	0.60 (0.30–1.21)	1.82 (1.12–2.95)
Repeated measurements model 1	1.05 (0.81–1.37)	0.89 (0.61–1.30)	0.75 (0.39–1.42)	1.64 (1.02–2.64)
**Stilbenes**				
Age and sex adjusted HR (95% CI)	1.12 (0.88–1.42)	1.09 (0.75–1.60)	0.72 (0.39–1.33)	1.23 (0.81–1.86)
Multivariable adjusted model 1	1.03 (0.18–1.38)	1.10 (0.79–1.52)	0.78 (0.38–1.60)	1.11 (0.62–1.96)
Repeated measurements model 1	0.98 (0.73–1.32)	1.18 (0.81–1.71)	0.81 (0.39–1.67)	1.05 (0.59–1.87)
**Other phenolic compounds**				
Age and sex adjusted HR (95% CI)	1.04 (0.84–1.28)	1.06 (0.77–1.45)	0.85 (0.50–1.46)	1.50 (1.03–2.18)
Multivariable adjusted model 1	1.04 (0.80–1.35)	0.95 (0.66–1.36)	0.62 (0.31–1.22)	1.59 (0.98–2.56)
Repeated measurements model 1	1.06 (0.85–1.32)	1.08 (0.76–1.53)	0.67 (0.34–1.30)	1.30 (0.79–2.14)

Abbreviations: CI, confidence interval; HR, hazard ratio; Q, quintile. All Cox regression models used age as the underlying time variable and were also stratified by age (five-year periods), recruitment period, marital status, and years of university education. Multivariable adjusted model 1: Additionally adjusted for energy intake (kcal/day), smoking status (never smoker, current smoker or former smoker), lifetime tobacco exposure (packs-years), passive smoking (yes/no), BMI (kg/m^2^) and the quadratic term, height (m), prevalent cardiovascular disease, cancer, diabetes, atrial fibrillation, dyslipidemia, hypertension and depression, (yes/no), family history of CVD and cancer (yes/no), physical activity (metabolic equivalents-h/week) (tertiles), TV watching time (hours/day). Repeated measurements analyses were adjusted for the same variables as model 1 with updated data on dietary variables at 10 years of follow-up (except for participants with a diagnosis of cancer, CVD or diabetes during follow-up, *n* = 652)

When we analyzed PC intake by classes, low flavonoids intake showed a significant increased risk of mortality only when non-cancer/non-CVD mortality were analyzed in those over 45 years in multivariable model 1 (HR: 1.75; 95% CI: 1.05–2.91; *p*-value: 0.03) and in repeated measurements (HR: 1.78; 95% CI: 1.09–2.94; *p*-value:0.02) (Table 4). On the other hand, we observed non-significant results in the risk for CVD mortality (HR: 0.70; 95% CI: 0.35–1.38) in association with a low flavonoid intake (Table 4), which was similar when participants younger than 45 years were included in the analyses (HR: 0.61; 95% CI: 0.35–1.45). Low lignan intake showed inconclusive results for all-cause mortality. In cause-specific mortality we observed a non-significant increased risk in cancer mortality for a low intake of lignans (HR: 1.29; 95% CI: 0.90–1.84) (Table 4). When participants younger than 45 years were included in the analysis of the effects of a low lignan intake, an elevated but statistically non-significant risk of non-cancer/non-CVD mortality was observed in multivariable model 1 (HR: 1.56; 95% CI: 0.99–2.46) and in the cumulative average analysis (HR: 1.35; 95% CI: 0.83–2.17) (Supplementary Table 2).

For low phenolic acids intake, participants over 45 years showed significant higher risks in the non-cancer/non-CVD mortality category when comparing Q1 vs. Q4-Q5 in repeated measurements model (HR: 1.64; 95% CI: 1.02–2.64; *p*-value: 0.04) and in multivariable model 1 (HR: 1.82; 95% CI: 1.12–2.65; *p*-value:0.01) (Table 4). When analyzing the whole sample (also those younger than 45 years) the group of phenolic acids was the only PC class in which the statistically significance was maintained in model 1 (HR: 1.65; 95% CI: 1.02–2.66; *p*-value:0.04) and in the repeated measurements analysis (HR: 1.61; 95% CI: 1.00-2.60; *p*-value:0.04), (Supplementary Table 2).

As a sensitivity analyses, deaths due to external causes were excluded (*n* = 17 in the sample of participants over 45 years, and *n* = 27 in the total sample). Statistically significant associations were consistently maintained for both total mortality and mortality due to causes other than cancer and CVD. Similar results were also obtained in the analyses by class when the consumption of PC that did not belong to the class under study was included as a confounding factor. Interactions between PC consumption and BMI, as well as sex, yielded no statistically significant results. Lastly, we did not observe any nonlinear relationships between total or class-specific PC intake and mortality.

## Discussion

In our analysis in the SUN cohort, when assessing the effects of a low PC intake (lowest versus the four upper quintiles) in subjects over 45 years old we observed a 32% relatively higher risk of all-cause mortality, 44% relatively higher risk of cancer mortality and 69% relatively higher risk of mortality from causes other than cancer or CVD. In contrast, CVD mortality showed a non-significant association with a low total PC intake.

Chocolate, coffee, and cherries were the primary sources of total PC linked to an increased risk of all-cause death as well as cancer and other cause specific mortality (non-cancer/non-CVD) at low intakes. These sources were also linked to a non-significant and not expected decreased risk in CVD mortality due to a low intake of total PC. When subjects under the age of 45 were included in the analysis, no significant differences in total PC intake and classes were detected. Perhaps because the protective effect of PC on pathological processes becomes more evident at advanced ages.

In Spain, some studies have focused on PC dietary intake and mortality. The landmark PREDIMED trial assessed the effect of a Mediterranean diet intervention in elderly at high CVD risk and found that higher intakes of total PC were associated with a 37% lower risk of all-cause mortality [12]. Moreover, another recent Spanish cohort study (ENRICA) found that higher intake of some specific PC subclasses of flavonoids and phenolic acids at baseline were associated with lower all-cause mortality with a relatively 20% lower risk [16] and the EPIC-Spain cohort also reported a protective association for some flavonoid subgroups on overall mortality [13]. Total PC intake and mortality have been scarcely evaluated in cohorts from other countries [32]. However, we found abundant evidence of the protective effect of flavonoid intake in reducing all-cause, cancer and CVD mortality risk in other cohorts with an L-shaped relationship instead of an inverse linear dose-response relationship [32, 33].

Our study observed higher cancer mortality risk for a reduced total PC and flavonoid intake, but the association was only statistically significant for total PC. Other studies in Spain [13, 16] and other countries with dietary patterns that traditionally contain PC-rich foods, such as Japan, did not find protective effects of PC intake on cancer mortality [34]. Additionally, a non-significant relationship between flavonoid intake and cancer mortality was observed in a recent meta-analysis of cohort studies but it only included four studies [35]. Results from two large US cohorts assessing the effect of phytoestrogens, such as isoflavones, lignans and coumarins on mortality, found no association with cancer mortality after following more than 75,980 women and 444,001 men for an average of 34 years [36]. Nevertheless, a study of the Danish Diet Cancer and Health Cohort showed that a flavonoid intake above quintile 1 was associated with a lower risk of cancer-related mortality but the mean intake per quintile was considerably higher than ours [37]. In an Iranian cohort, the Golestan cohort study, an association between two flavonoid subgroups, isoflavonoids and dihydrochalcones, and cancer mortality comparing extreme categories of intake was observed [11]. Current evidence appears to be inconclusive concerning total PC, flavonoid intake and cancer mortality [38]. It may be crucial to be more comprehensive in analyzing the effects of total PC intake by cancer type [39]. Extending research to other PC classes in addition to flavonoids should also be considered because according to our results, total PC intake showed a stronger protective association than flavonoids intake alone.

Regarding CVD mortality, we did not find significant results for total PC intake or for specific PC classes intake. There is abundant evidence about the protective role of a PC-rich diet against the incidence of CVD [40, 41], which has also been previously demonstrated in our cohort using fatal and non-fatal cases, here we only assessed fatal cases [42, 43]. Some studies found that higher intake of phenolic acids [16], flavonoids [11] and some specific subclasses of other PC classes [16] reduced the risk of CVD mortality when comparing extreme intake categories. In addition, another Spanish cohort has recently shown that increased total PC intakes reduced the risk of CVD mortality [16]. However, considering our results, the effect of a low intake of total PC and by class in our cohort has not been conclusive when examining CVD mortality. In our cohort, the mortality rate from CVD is relatively low compared to other causes, possibly due to the relatively young age of participants and their overall healthy lifestyles. We may lack the statistical power to observe significant differences in mortality because of this reason.

We found the highest increases in mortality risks due to a low total PC intake, flavonoid and phenolic acids in other cause-specific mortality category (non-cancer/non-CVD), where the main causes were respiratory and neurodegenerative diseases. Recent literature suggests that dietary PC intake may be related to certain respiratory health benefits. Some studies have explored the relationship between flavonoid intake and a reduced risk of developing asthma and better lung function in people with asthma due to antioxidant, anti-allergic immune-modulating activities of PC [44]. Also, PC intake has shown beneficial association with chronic obstructive pulmonary disease (COPD). A dose-response meta-analysis of observational studies suggested that high intake of PC-rich foods, such as fruit and vegetables, might be related to a lower risk of developing COPD [45]. Strong inverse associations were found between anthocyanin intake and age-related decline in lung function [46] and between total flavonoid intake and COPD in current smokers and former smokers independent of dietary and nondietary risk factors [47]. Additionally, flavonoids and phenolic acids have been suggested to have antimicrobial and anti-infectious properties that may be helpful in the prevention of respiratory infections [48, 49]. This evidence is in line with our result and points to a protective and mitigating role of PC intake in respiratory diseases that can lead to lower respiratory mortality in our cohort. However, it is important to note that evidence in this area may be limited or inconclusive and further research is needed. Neurodegenerative diseases, Parkinson’s disease, Alzheimer’s disease, and other forms of age-related dementia were the second leading cause of other mortality category. The connection between dietary PC and neurodegenerative diseases has been recently studied, although the evidence is currently limited, and more research is required [50–53]. To our knowledge, no study has assessed the effect of phenolic acid intake on neurodegenerative mortality so far.

In our cohort, higher mortality associated with a low intake of PC may be linked to the increase of non-CVD mortality in subjects with lower intakes. The underlying mechanisms by which optimal PC intake may contribute to higher life expectancy are still unclear. According to several studies, PC can reduce age-related processes such as oxidative stress, inflammation, impaired proteostasis and cellular senescence, which are linked to aging-related diseases. Also, PC have shown anticarcinogenic effects. As such, several reviews have summarized the potential chemo preventive mechanisms of PC such as their ability to modulate carcinogen metabolism (e.g., phase I and II metabolic enzymes), regulate inflammatory pathways, inhibit cell proliferation, induce tumor cell apoptosis and modulate anticancer responses to chemotherapy [54]. In addition, an adequate intake of PC could play a relevant role in the prevention of diseases through other mechanisms, such as the neutralization of free radicals and reduction of oxidative stress, which can in turn damage cells and DNA. PC are also able to reduce chronic inflammation [55, 56]. Furthermore, PC may act as a prebiotic, promoting intestinal health and thus immune function [57]. In this regard, PC have also shown antimicrobial effects that can help fight bacterial and viral infections [48]. Nevertheless, the molecular mechanisms and specificity of action of phytochemicals such as PCs constitute a complex field of research [55].

This study presented some limitations. One of them may be linked to the estimation of PC intake, although we used the most recent and complete database available (Phenol-explorer version 3.6), since not all FFQ-derived foods are included in the database (e.g., honey) and the FFQ does not include all PC-rich foods (e.g., spices, herbs, or some seeds). Another aspect to consider is that an FFQ does not distinguish between foods in complete detail, which may influence the PC content of different foods (e.g., cocoa percentage of different varieties of chocolate, types of coffee or tea). Nevertheless, some validation studies have concluded that FFQ are suitable for estimating PC intake [26]. In addition, bioavailability after consumption was not taken into consideration in this study and it may show some degree of inter-person variability. Also, PC food content can be influenced by external conditions such as storage, ripeness, processing and plant variety, that may affect the PC actions [8]. Lastly, we may not have been able to fully capture changes in PC intake between baseline FFQ and 10-year follow-up. The potential measurement error more likely contributed to attenuate the estimates of relative risk that we presented here. However, this study has several strengths, such as adjustments for comprehensive and validated data on possible confounders, its prospective design, a high retention rate of the cohort (93.4%) and comprehensive ascertainment of deaths by the Spanish National Death Index. Furthermore, by accounting for dietary changes throughout a 10-year follow-up period, discrepancies in dietary measurements were reduced, allowing for a more accurate assessment of food intake over time. This study was conducted in a Mediterranean country, which may be advantageous in capturing the impact of PC-rich foods such as olive oil or red wine, both hallmarks of the Mediterranean dietary pattern. Future research of possible associations between dietary PC and mortality should be combined with studies assessing exposure using additional approaches, such as biomarkers of consumption, metabolism, and excretion [58].

## Conclusion

Our findings reveal that in subjects over 45-year-old a reduced total PC intake is associated with a significant higher risk of mortality from causes other than CVD. Among PC classes, a low intake of flavonoids and phenolic acids showed a significant increase in mortality (78% and 75% relatively higher risk respectively) in non-cancer/CVD mortality. Cherries, chocolate, apples and pears, olives, and coffee, were the major sources of variability in total PC intake. For flavonoids intake, chocolate and cherries were the main sources of variability whereas coffee account for the largest part of variability in phenolic acids intake.

## Electronic supplementary material

Below is the link to the electronic supplementary material.

Supplementary Material 1

## Data Availability

The data that support the findings of this study are available from the SUN Project upon reasonable request at sun@unav.es.
