# Supplementary material for: Effect of dietary phenolic compounds intake on mortality in the “Seguimiento Universidad De Navarra” (SUN) Mediterranean cohort
Source: Eur J Nutr. 2025 Jan 24;64(2):70. doi: 10.1007/s00394-025-03581-5 (PMC11761832; doi:10.1007/s00394-025-03581-5)
Supplement: Supplementary file 1 — Supplementary Material 1 [file 394_2025_3581_MOESM1_ESM.docx]

Supplemental table 1. Hazard ratio (HR) and 95% confidence intervals (CI) of all-cause mortality cases according to quintiles of total phenolic compounds intake and classes using the lowest quintile as the reference category.

|  | Quintiles of intake | | | | | |
| --- | --- | --- | --- | --- | --- | --- |
|  | 1 | 2 | 3 | 4 | 5 | p for trend |
| **Total phenolic compounds** |  |  |  |  |  |  |
| Median intake (mg/d) | 435.4 | 604.2 | 734.7 | 888.5 | 1180.9 |  |
| N | 3634 | 3634 | 3634 | 3634 | 3634 |  |
| Cases | 79 | 98 | 105 | 111 | 151 |  |
| Person-years | 47455 | 47962 | 47384 | 46879 | 46648 |  |
| Age and sex adjusted HR (95% CI) | 1 (ref.) | 0.82 (0.61-1.11) | 0.83 (0.66-1.19) | 0.70 (0.52-0.94) | 0.77 (0.58-1.01) | 0.07 |
| Multivariable adjusted model 1 | 1 (ref.) | 0.85 (0.61-1.18) | 0.95 (0.68-1.31) | 0.78 (0.56-1.08) | 0.85 (0.62-1.16) | 0.36 |
| Repeated measurements model 1 | 1 (ref.) | 0.84 (0.60-1.17) | 0.94 (0.68-1.31) | 0.72 (0.52-1.00) | 0.89 (0.64-1.20) | 0.43 |
| **Flavonoids** |  |  |  |  |  |  |
| Median intake (mg/d) | 191.6 | 302.7 | 389.0 | 499.0 | 723.9 |  |
| N | 3634 | 3634 | 3634 | 3634 | 3634 |  |
| Cases | 84 | 98 | 110 | 122 | 130 |  |
| Person-years | 48191 | 47513 | 47686 | 46546 | 46392 |  |
| Age and sex adjusted HR (95% CI) | 1 (ref.) | 0.90 (0.67-1.20) | 0.85 (0.63-1.28) | 0.85 (0.64-1.34) | 0.74 (0.56-0.98) | 0.04 |
| Multivariable adjusted model 1 | 1 (ref.) | 0.89 (0.64-1.24) | 1.00 (0.73-1.37) | 0.93 (0.68-1.29) | 0.81 (0.60-1.11) | 0.21 |
| Repeated measurements model 1 | 1 (ref.) | 0.84 (0.60-1.16) | 1.01 (0.73-1.40) | 0.84 (0.60-1.16) | 0.84 (0.62-1.16) | 0.29 |
| **Lignans** |  |  |  |  |  |  |
| Median intake (mg/d) | 1.2 | 1.7 | 2.1 | 2.5 | 3.4 |  |
| N | 3634 | 3634 | 3634 | 3634 | 3634 |  |
| Cases | 86 | 97 | 111 | 122 | 128 |  |
| Person-years | 49212 | 48461 | 47588 | 46279 | 44789 |  |
| Age and sex adjusted HR (95% CI) | 1 (ref.) | 0.84 (0.63-1.12) | 0.86 (0.65-1.15) | 0.71 (0.53-0.94) | 0.65 (0.49-0.86) | 0.002 |
| Multivariable adjusted model 1 | 1 (ref.) | 0.92 (0.67-1.26) | 0.91 (0.67-1.24) | 0.84 (0.61-1.15) | 0.79 (0.57-1.09) | 0.13 |
| Repeated measurements model 1 | 1 (ref.) | 0.99 (0.71-1.37) | 0.97 (0.70-1.34) | 0.85 (0.61-1.17) | 0.80 (0.59-1.11) | 0.09 |
| **Phenolic acids** |  |  |  |  |  |  |
| Median intake (mg/d) | 147.8 | 222.4 | 286.4 | 353.1 | 484.7 |  |
| N | 3634 | 3634 | 3634 | 3634 | 3634 |  |
| Cases | 71 | 93 | 119 | 121 | 140 |  |
| Person-years | 46543 | 47409 | 46834 | 47561 | 47982 |  |
| Age and sex adjusted HR (95% CI) | 1 (ref.) | 0.78 (0.57-1.07) | 1.02 (0.76-1.38) | 1.02 (0.79-1.37) | 0.82 (0.61-1.10) | 0.45 |
| Multivariable adjusted model 1 | 1 (ref.) | 0.89 (0.63-1.25) | 1.15 (0.83-1.61) | 1.13 (0.81-1.57) | 0.91 (0.67-1.26) | 0.70 |
| Repeated measurements model 1 | 1 (ref.) | 0.77 (0.55-1.09) | 1.21 (0.87-1.68) | 1.02 (0.73-1.42) | 0.91 (0.66-1.26) | 0.86 |
| **Stilbenes** |  |  |  |  |  |  |
| Median intake (mg/d) | -0.0 | 0.1 | 0.4 | 0.8 | 3.6 |  |
| N | 3634 | 3634 | 3634 | 3634 | 3634 |  |
| Cases | 84 | 76 | 84 | 108 | 192 |  |
| Person-years | 47914 | 46257 | 46258 | 46784 | 49115 |  |
| Age and sex adjusted HR (95% CI) | 1 (ref.) | 0.84 (0.61-1.14) | 0.82 (0.61-1.11) | 0.90 (0.67-1.20) | 1.00 (0.77-1.30) | 0.19 |
| Multivariable adjusted model 1 | 1 (ref.) | 0.97 (0.68-1.39) | 0.83 (0.60-1.19) | 0.93 (0.66-1.30) | 0.98 (0.72-1.34) | 0.59 |
| Repeated measurements model 1 | 1 (ref.) | 1.03 (0.72-1.49) | 0.86 (0.60-1.26) | 0.94 (0.66-1.33) | 0.97 (0.73-1.37) | 0.72 |
| **Other phenolic compounds** |  |  |  |  |  |  |
| Median intake (mg/d) | 14.2 | 25.4 | 34.0 | 45.2 | 72.5 |  |
| N | 3634 | 3634 | 3634 | 3634 | 3634 |  |
| Cases | 106 | 95 | 105 | 106 | 132 |  |
| Person-years | 48533 | 48103 | 47295 | 46054 | 46344 |  |
| Age and sex adjusted HR (95% CI) | 1 (ref.) | 0.83 (0.63-1.09) | 0.97 (0.74-1.28) | 0.90 (0.70-1.21) | 0.96 (0.74-1.25) | 0.83 |
| Multivariable adjusted model 1 | 1 (ref.) | 0.91 (0.66-1.26) | 1.02 (0.75-1.39) | 1.05 (0.77-1.44) | 1.09 (0.81-1.47) | 0.32 |
| Repeated measurements model 1 | 1 (ref.) | 0.96 (0.69-1.31) | 1.03 (0.75-1.42) | 1.01 (0.74-1.40) | 1.06 (0.79-1.43) | 0.57 |

*p< 0.05. Abbreviatures: CI, confidence interval; HR, hazard ratio; Q, quintile. All Cox regression models used age as the underlying time variable and were also stratified by age (five-year periods), recruitment period, marital status, and years of university education. Multivariable adjusted model 1: Additionally adjusted for energy intake (kcal/day), smoking status (never smoker, current smoker or former smoker), lifetime tobacco exposure (packs-years), passive smoking (yes/no), BMI (kg/m^2^) and the quadratic term, height (m), prevalent cardiovascular disease, cancer, diabetes, atrial fibrillation, dyslipidemia, hypertension and depression, (yes/no), family history of CVD and cancer (yes/no), physical activity (metabolic equivalents-h/week) (tertiles), TV watching time (hours/day). Repeated measurements analyses were adjusted for the same variables as multivariable adjusted model with updated data on dietary variables at 10 years of follow-up (except for participants with a diagnosis of cancer, CVD or diabetes during follow-up, n=909).

Supplemental table 2. Hazard ratio (HR) and 95% confidence intervals (CI) of all-cause and cause-specific mortality according to a low intake (Q1) vs. a moderate-high intake (Q2-Q4) using the four upper quintiles as the reference category.

|  | **All-cause mortality** | **Cancer mortality** | **CVD mortality** | **Non-cancer, non-CVD**  **mortality** |
| --- | --- | --- | --- | --- |
|  | Q1 vs Q2-5 (Reference) | Q1 vs Q2-5 (Reference) | Q1 vs Q2-5 (Reference) | Q1 vs Q2-5 (Reference) |
| Number of cases/person-years | 544/236,328.64 | 275/233,319.96 | 101/231,485.32 | 168/227,949.59 |
| **Total phenolic compounds** |  |  |  |  |
| Age-sex adjusted HR (95% CI) | 1.18 (0.92 - 1.50) | 1.23 (0.88 - 1.72) | 0.83 (0.43 - 1.61) | 1.27 (0.83 - 1.95) |
| Multivariable adjusted model 1 | 1.16 (0.89 - 1.52) | 1.19 (0.82 - 1.73) | 0.90 (0.43 - 1.90) | 1.32 (0.81 - 2.15) |
| Repeated measurements model 1 | 1.18 (0.90 - 1.54) | 1.19 (0.83 - 1.72) | 0.76 (0.36 - 1.60) | 1.37 (0.85 - 2.21) |
| **Flavonoids** |  |  |  |  |
| Age-sex adjusted HR (95% CI) | 1.14 (0.90 - 1.45) | 1.23 (0.89 - 1.71) | 0.92 (0.50 - 1.70) | 1.12 (0.73 - 1.71) |
| Multivariable adjusted model 1 | 1.09 (0.84 - 1.42) | 1.17 (0.82 - 1.68) | 0.93 (0.46 – 1.87) | 1.07 (0.66 - 1.77) |
| Repeated measurements model 1 | 1.13 (0.87 - 1.48) | 1.14 (0.81 - 1.63) | 0.71 (0.35 - 1.45) | 1.33 (0.82 - 2.15) |
| **Lignans** |  |  |  |  |
| Age-sex adjusted HR (95% CI) | 1.18 (0.93 - 1.50) | 1.02 (0.72 - 1.45) | 1.18 (0.67 - 2.08) | 1.45 (0.97 - 2.17) |
| Multivariable adjusted model 1 | 1.14 (0.88 - 1.48) | 1.00 (0.69 - 1.46) | 0.99 (0.51 - 1.93) | 1.56 (0.99 - 2.46) |
| Repeated measurements model 1 | 1.11 (0.84 - 1.45) | 1.08 (0.74 - 1.56) | 1.17 (0.61 - 2.24) | 1.35 (0.83 - 2.17) |
| **Phenolic acids** |  |  |  |  |
| Age-sex adjusted HR (95% CI) | 1.06 (0.82 - 1.36) | 0.94 (0.65 - 1.36) | 0.74 (0.37 - 1.49) | 1.46 (0.97 - 2.21) |
| Multivariable adjusted model 1 | 1.00 (0.76 - 1.33) | 0.87 (0.58 - 1.30) | 0.51 (0.22 - 1.19) | 1.65 (1.02 - 2.66) |
| Repeated measurements model 1 | 1.05 (0.81 - 1.38) | 0.85 (0.57 - 1.27) | 0.51 (0.22 - 1.17) | 1.61 (1.00 - 2.60) |
| **Stilbenes** |  |  |  |  |
| Age-sex adjusted HR (95% CI) | 1.11 (0.88 - 1.41) | 0.99 (0.71 - 1.39) | 1.00 (0.59 - 1.92) | 1.40 (0.94 - 2.09) |
| Multivariable adjusted model 1 | 1.05 (0.79 - 1.38) | 1.00 (0.68 - 1.48) | 0.97 (0.48 - 1.95) | 1.15 (0.68 – 1.93) |
| Repeated measurements model 1 | 1.02 (0.84 - 1.46) | 1.01 (0.69 - 1.47) | 1.04 (0.52 - 2.10) | 1.21 (0.72 – 2.04) |
| **Other phenolic compounds** |  |  |  |  |
| Age-sex adjusted HR (95% CI) | 1.04 (0.84 - 1.28) | 1.04 (0.76 - 1.40) | 0.75 (0.43 - 1.31) | 1.21 (0.84 - 1.75) |
| Multivariable adjusted model 1 | 0.97 (0.75 - 1.24) | 0.98 (0.70 - 1.38) | 0.56 (0.28 - 1.13) | 1.18 (0.75 - 1.86) |
| Repeated measurements model 1 | 0.97 (0.76 - 1.25) | 1.07 (0.76 - 1.49) | 0.60 (0.30 - 1.19) | 1.03 (0.64 - 1.65) |

Abbreviations: CI, confidence interval; HR, hazard ratio; Q, quintile. All Cox regression models used age as the underlying time variable and were also stratified by age (five-year periods), recruitment period, marital status, and years of university education. Multivariable adjusted model 1 : Additionally adjusted for energy intake (kcal/day), smoking status (never smoker, current smoker or former smoker), lifetime tobacco exposure (packs-years), passive smoking (yes/no), BMI (kg/m^2^) and the quadratic term, height (m), prevalent cardiovascular disease, cancer, diabetes, atrial fibrillation, dyslipidemia, hypertension and depression, (yes/no), family history of CVD and cancer (yes/no), physical activity (metabolic equivalents-h/week) (tertiles), TV watching time (hours/day). Repeated measurements analyses were adjusted for the same variables as model multivariable adjusted model with updated data on dietary variables at 10 years of follow-up (except for participants with a diagnosis of cancer, CVD or diabetes during follow-up, n=909).
